# Combined virgin coconut oil and tocotrienol-rich fraction protects against bone loss in osteoporotic rat model

**DOI:** 10.14202/vetworld.2019.2052-2060

**Published:** 2019-12-25

**Authors:** Mohd Maaruf Abdul Malik, Faizah Othman, Farida Hussan, Ahmad Nazrun Shuid, Qodriyah Mohd Saad

**Affiliations:** 1 Department of Anatomy, Faculty of Medicine, Universiti Kebangsaan Malaysia Medical Centre, Jalan Yaacob Latif, 56000 Cheras, Kuala Lumpur, Malaysia; 2 Centre of Preclinical Science Studies, Faculty of Dentistry, Universiti Teknologi MARA, Sungai Buloh Campus, Jalan Hospital, 47000 Sungai Buloh, Selangor, Malaysia; 3 Department of Anatomy, Human Biology Division, School of Medicine, International Medical University, Jalan Jalil Perkasa 19, Bukit Jalil, 57000 Kuala Lumpur, Malaysia; 4 Department of Pharmacology, Faculty of Medicine, Universiti Kebangsaan Malaysia Medical Centre, Jalan Yaacob Latif, 56000 Cheras, Kuala Lumpur, Malaysia

**Keywords:** bone loss, osteoporosis, ovariectomized rat, ovariectomy, tocotrienol-rich fraction, virgin coconut oil

## Abstract

**Background and Aim:**

Both virgin coconut oil (VCO) and tocotrienol-rich fraction (TRF) are rich in antioxidants and may protect the bone against bone loss induced by ovariectomy and high-fat diet. The study aimed to determine the protective effects of combined therapy of VCO and TRF on osteoporosis in ovariectomized (OVX) rat fed with high-fat diet.

**Materials and Methods:**

Thirty-six female Sprague-Dawley rats were divided into six groups: Sham-operated (SHAM), OVX control, OVX and given Premarin at 64.5 µg/kg (OVX+E2), OVX and given VCO at 4.29 ml/kg (OVX+V), OVX and given TRF at 30 mg/kg (OVX+T), and OVX and given a combination of VCO at 4.29 ml/kg and TRF at 30 mg/kg (OVX+VT). Following 24 weeks of treatments, blood and femora samples were taken for analyses.

**Results:**

There were no significant differences in serum osteocalcin levels between the groups (p>0.05), while serum C-terminal telopeptide of Type I collagen levels of the OVX+VT group were significantly lower than the other groups (p<0.05). The dynamic bone histomorphometry analysis of the femur showed that the double-labeled surface/bone surface (dLS/BS), mineral apposition rate, and bone formation rate/BS of the OVX+E2, OVX+T, and OVX+VT groups were significantly higher than the rest of the groups (p<0.05).

**Conclusion:**

A combination of VCO and TRF has the potential as a therapeutic agent to restore bone loss induced by ovariectomy and high-fat diet.

## Introduction

Osteoporosis is known as a silent metabolic bone disease characterized by low bone mass and microarchitecture damage, resulting in increased risk of fractures. It is caused by reduced osteoblastic activity and increased osteoclastic activity. It commonly occurs in elderly women due to lack of estrogen following menopause [1]. Osteoporosis induced by estrogen deficiency could be further aggravated by unhealthy diet intake. The study has shown that cholesterol is one of the factors involved in stimulating osteoclast formation and survival [2] by promoting interleukin-1 production. Intake of repeatedly heated palm oil may be detrimental to the bone structure of ovariectomized (OVX) rat model [3]. A combination of these two unhealthy diets has been shown to worsen the bone deterioration caused by ovariectomy [4]. The effects of the unhealthy diet were thought to be related to oxidative stress [5]. Estrogen has antioxidant effects and was positively correlated with the levels of plasma antioxidants and antioxidant enzymes [6]. Estrogen also enhanced the expression of glutathione peroxidase (GPX), an enzyme that degrades hydrogen peroxide [7]. Therefore, estrogen deficiency caused reduction in GPX, thus predisposing the bones to hydrogen peroxide. The lack of estrogen also reduces its protective effect against oxidative stress [8]. A previous study showed that estrogen deficiency stimulated bone loss, which in turn contributed to the development of osteoporosis [9].

Estrogen replacement therapy (ERT) is the primary treatment and prevention of postmenopausal osteoporosis [10]. Either estrogen alone or in combination with progesterone may prevent bone loss after menopause. These therapies are effective in preventing bone loss but do not reverse the bone loss. Long-term ERT may also increase the risk of breast cancer, coronary heart disease, stroke, and dementia [11]. Due to these serious side effects of ERT, researchers are finding alternative treatment [12] that is effective but has fewer side effects. One of the most popular natural products is virgin coconut oil (VCO), which has been extracted directly from fresh, mature coconut kernel without undergoing a refined process. This preserves the essential biologically active compounds in the oil such as tocotrienols, polyphenols, and tocopherols, which possess antioxidant properties [13]. Dietary supplementations of VCO had increased the antioxidant properties in rats [14]. VCO supplementation provided protection against bone loss in osteoporosis [15]. VCO has also been accounted to have anticancer, antimicrobial, and anti-inflammatory properties [16-18]. Furthermore, the effects of tocotrienols on bone parameters using different osteoporosis models such as ovariectomy [19], steroid-induced [20], and nicotine-induced [21] models have been conducted by researchers. All the studies showed that tocotrienols offered protection against bone loss in osteoporosis models. The mechanism of protection is related to its antioxidant properties.

Combined effects of VCO and tocotrienol-rich fraction (TRF) on bone loss in osteoporosis have not been explored yet and may be beneficial with the added oxidative stress of unhealthy diets. Therefore, the current study was designed to determine the effects of VCO and TRF, individually and in combination, on the bone parameters of OVX rat fed with high cholesterol diet and repeatedly heated palm oil.

## Materials and Methods

### Ethical approval

The research project was conducted from May 2014 to August 2016 in Universiti Kebangsaan Malaysia Medical Centre (UKMMC), Cheras, Kuala Lumpur, Malaysia. This research was approved by the Research and Ethical Committee, Faculty of Medicine, UKMMC (FP/ANAT/2014/FAIZAH).

### Experimental design

Thirty-six female Sprague-Dawley rats, weighing between 250 and 300 g, were obtained from the Laboratory Animals Resource Unit, Faculty of Medicine, UKMMC. The animals were allowed 1-week of acclimatization during which they were fed on commercial rat chow (Gold Coin, Klang, Selangor, Malaysia). The rats were randomized into six groups of six animals each: Sham-operated (SHAM), OVX-control, OVX and given Premarin 64.5 µg/kg (OVX+E2), OVX and given VCO 4.29 ml/kg (OVX+V), OVX and given TRF 30 mg/kg (OVX+T), and OVX and given combination of VCO 4.29 ml/kg and TRF 30 mg/kg (OVX+VT). The rats were housed one per cage in stainless-steel cages at 27±2°C with adequate ventilation and 12-h light/dark cycle. After 2 weeks of ovariectomy, the SHAM group was fed with standard rat chow while the OVX rats were given high cholesterol diet mixed with repeatedly heated palm oil. All the rats were allowed free access to food and tap water *ad libitum*. The treatments were administered to the OVX+E2, OVX+V, OVX+T, and OVX+VT groups daily through oral gavage for 24 weeks. The food intake and body weights were recorded daily and weekly, respectively. Blood was drawn to measure bone biochemical markers (osteocalcin [OC] and C-terminal telopeptide of Type I collagen [CTX]) in the serum before and after treatments. After 24 weeks, the rats were sacrificed, and the left femora were dissected and prepared for dynamic histomorphometric studies. All animal management and procedures were performed in accordance with the recommended guidelines for the care and use of laboratory animals. Care was taken to minimize discomfort, distress, and pain to the animals.

### Ovariectomy

Ovariectomy was carried out under ketamil: ilium xylazil-20 (Troy Laboratories PTY, Australia) in 1:1 ratio, which was injected intramuscularly with the dose of 0.1 ml/100 g body weight of the rats. Once anesthetized, the furs on the abdomen were shaved. A vertical incision was made in the abdomen using a sterilized sharp knife, and both ovaries were identified. The fallopian tubes were ligated before removing the ovaries. The muscular layer under the skin was stitched up by catgut suture (Serafit, Germany) while the outer layer of skin was stitched with Mersilk Thread (Seralon, Serag Wiessner, Germany). The abdomen of the SHAM group was opened, whereby their ovaries were exposed and carefully manipulated but left intact [22]. The rats were left recuperating for 2 weeks before commencing the treatment.

### Preparation of high cholesterol diet and repeatedly heated palm oil

Palm oil (Cap Buruh, Lam Soon Edible Oils, Kuala Lumpur, Malaysia) was purchased from a local manufacturer. The palm oil was heated 5 times. Briefly, 2.5 L of fresh palm oil was used to fry 1 kg of sweet potato slices in a stainless-steel wok. The temperature of the heated oil was maintained at 180°C for 10 min. Then, the oil was cooled down at room temperature for 5 h. The whole frying process was repeated 4 more times with a new batch of sweet potatoes without adding any fresh oil. Then, the 5 times heated oil (5HO) was collected to prepare the special diet by mixing 15% (w/w) of 5HO to the high cholesterol diet. The diets were made into pellets and dried in the oven at 70°C overnight. The oil: high cholesterol diet ratio represents the average amount of daily oil intake in humans [23].

### Preparation of VCO, TRF, and Premarin

The VCO used in this study was purchased from Bio-Asli Sdn. Bhd., Sungai Besar, Selangor, Malaysia. The VCO was administered through oral gavage using a cannula needle at a dose of 4.29 ml/kg body weight of rats for 24 weeks. The dose was equivalent to VCO given to humans for alternative therapy, which was three tablespoons or equal to 45 ml/day [24].

TRF was prepared by Carotech (Tocomin, Selangor, Malaysia), consisting of alpha-tocotrienol (37.2%), gamma-tocotrienol (39.1%), and delta-tocotrienol (22.6%). It was diluted in olive oil (Bertolli Classico, Italy) and given daily through oral gavages at the dose of 30 mg/kg body weight of rats for 24 weeks. This dose was roughly equivalent to 3 mg/kg in humans, or 210 mg for a 70 kg man.

Estrogen dose given in this study was 64.5 µg/kg. Each Premarin tablet containing 0.625 mg of conjugated estrogens was crushed and dissolved in 20 ml of distilled water. The solution was mixed using a magnetic stirrer until homogenous. Then, Premarin solution was stored in the refrigerator at 4°C. The Premarin was given daily through oral gavage at the dose of 0.2 ml/100 g body weight of rats for 24 weeks.

### Blood collection and bone sampling

For the biochemical study, blood samples were collected at the beginning and after 24 weeks of treatment from the retro-orbital vein under diethyl ether anesthesia. After leaving the blood at room temperature for 3 h, the blood was centrifuged at 3000 rpm for 10 min and the serum was stored at −70°C until further use.

Following 24 weeks of treatment, the rats were anesthetized with diethyl ether and sacrificed humanely by cervical dislocation. The left femora were dissected, and adhering muscles were cleansed before fixing in 10% formalin.

### Biochemical markers parameter (OC and CTX)

Bone biochemical markers of OC and CTX were evaluated before and after the treatment by enzyme-linked immunosorbent assay (ELISA’s) machine (Leica CTR. MIC, Germany) using Rat-Mid OC ELISA kit (Nordic Biosciences, IDS, UK) and RatLaps^™^ CTX-1 ELISA kit (Nordic Biosciences, IDS, UK), respectively.

### Dynamic histomorphometric bone parameter (single-labeled surface/bone surface [sLS/BS], double-labeled surface/BS [dLS/BS], mineralized surface/BS [MS/BS], mineral apposition rate [MAR], and bone formation rate/BS [BFR/BS])

After fixation, the bones were cut sagittally at mid-shaft using a rotary electronic saw (Black & Decker, USA). The distal left femora were then cut into half longitudinally and subsequently dehydrated in graded concentrations of ethanol. The femora were embedded in polymer methyl methacrylate medium according to the manufacturer’s instructions (Osteo-Bed Bone Embedding Kit; Polysciences, USA). Then, the samples were sectioned at 7 µm thickness using a Manual Rotary Microtome (Model 2235, Leica, Germany).

For dynamic parameters, undecalcified and unstained bones were analyzed using an image analyzer with Pro-Plus 5.0 software (Media Cybernetics, Silver Spring, MD, USA) that was connected to fluorescence microscope (Nikon Eclipse 80 µ, Japan). Dynamic parameters were measured using double fluorescent labeling technique by intraperitoneal injection of 20 mg/kg calcein to the rats at 7 days and 2 days before they were sacrificed. The basic measurements for dynamic parameters were sLS/BS, %, dLS/BS, %, MS/BS, %, MAR, µm/day, and BFR/BS, µm^3^/µm^2^/day.

All dynamic measurements were carried out randomly at the metaphyseal region of the distal femora, which was located between 3 mm and 7 mm from the lowest point of the growth plate and 1 mm from the lateral cortex, excluding endocortical region [25]. The selected area was the secondary spongiosa area, which is rich in high-turnover trabecular bone. Trabecular bone was chosen because its remodeling process is more dynamic than cortical bone.

### Statistical analysis

The Kolmogorov-Smirnov test was used as a normality test. The paired-sample t-test was carried out to compare the same group before and after treatment. For normally distributed data, one-way analysis of variance followed by Tukey’s honestly significant difference *post hoc* test was utilized for comparison between treatment groups while Kruskal–Wallis and Mann–Whitney tests were used for data that were not normally distributed. Statistical analysis was performed using the Statistical Package for the Social Sciences software version 22.0 (SPSS Inc., Chicago, IL, USA). The results were presented as mean values±standard error of the mean. The statistical differences were considered significant at p<0.05.

## Results

### Mean daily food intake

**Figure-1:**
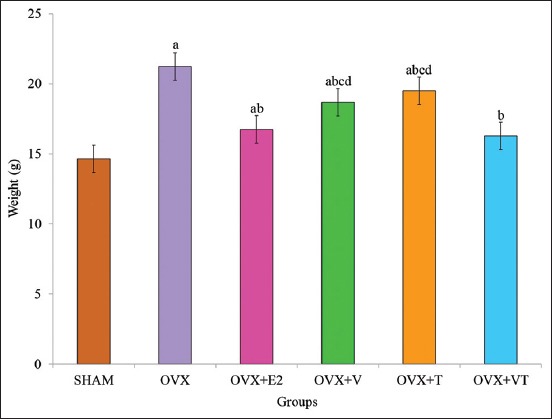
Mean daily food intake of all groups during the study treatment. Data are expressed as mean±standard error of the mean. (a) Significant difference compared to sham-operated group, (b) significant difference compared to ovariectomized group, (c) significant difference compared to ovariectomized with Premarin group, (d) significant difference compared to ovariectomized with virgin coconut oil and tocotrienol-rich fraction group.

Figure-1 shows the mean daily food intake of all groups throughout the study treatment. The results showed that the OVX, OVX+E2, OVX+V, and OVX+T groups had significantly higher mean daily food intake compared to the SHAM group (p<0.05). All the OVX treated groups, OVX+E2, OVX+V, OVX+T, and OVX+VT groups, showed significant lower food consumption compared to the negative control, OVX group (p<0.05). Among the treated groups, both OVX+V and OVX+T groups had significantly higher mean daily food intake compared to the OVX+E2 and OVX+VT groups (p<0.05).

### Weight gain

**Figure-2:**
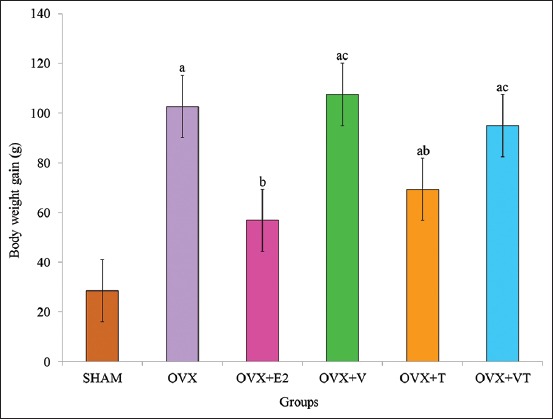
The body weight gain for all groups after 24-weeks study. Data presented as mean±standard error of the mean. (a) Significant difference compared to sham-operated group, (b) significant difference compared to ovariectomized group, (c) significant difference compared to ovariectomized with Premarin group.

The body weight gain for all groups after 24-week study is shown in Figure-2. The body weight gain of the OVX, OVX+V, OVX+T, and OVX+VT groups was significantly higher than the control, SHAM group (p<0.05). The weight gain of the OVX+E2 and OVX+T groups was significantly lower than that of the negative control, OVX group (p<0.05). Among the treated groups, both OVX+V and OVX+VT groups had significantly higher body weight gain compared to the positive control, OVX+E2 group (p<0.05).

### Serum OC

**Figure-3:**
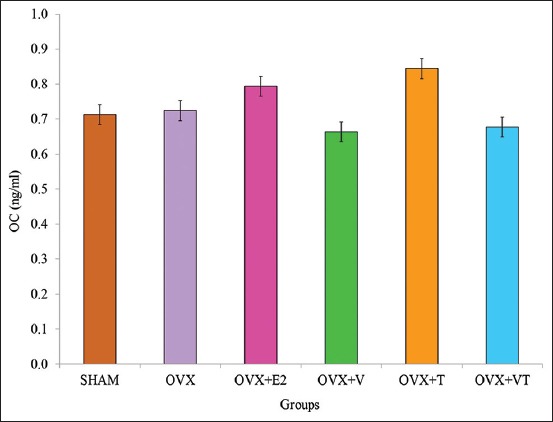
Post-treatment serum osteocalcin levels for all groups.

Figure-3 shows post-treatment serum OC levels for all groups. There was no significant difference in serum OC levels among the groups (p>0.05).

### Serum CTX

**Figure-4:**
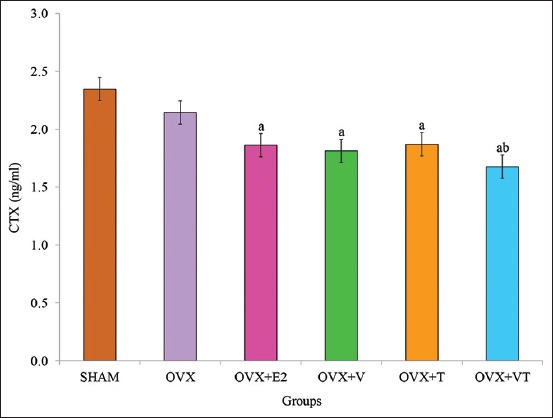
Post-treatment serum C-terminal telopeptide levels for all groups. (a) Significant difference compared to sham-operated group, (b) significant difference compared to ovariectomized group.

Figure-4 shows post-treatment serum CTX levels for all groups. After 24 weeks of treatment, the serum CTX levels in the OVX+E2, OVX+V, OVX+T, and OVX+VT groups were significantly lower compared to the control, SHAM group (p<0.05). Among the OVX groups, the post-treatment CTX level of the OVX+VT group was significantly lower than the negative control, OVX group (p<0.05).

### sLS/BS

**Figure-5:**
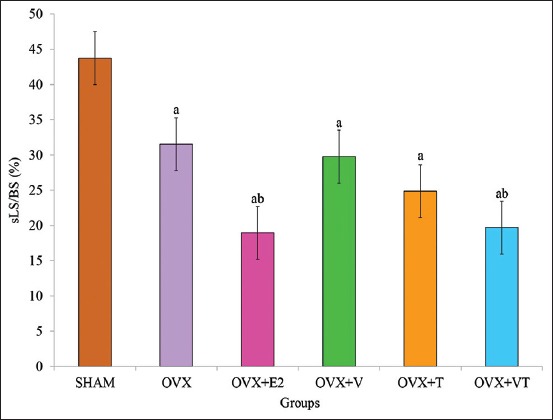
Single-labeled surface/bone surface. Data are expressed as mean±standard error of the mean. SHAM: Sham-operated; OVX: Ovariectomized; OVX+E2: Ovariectomized with Premarin; OVX+V: Ovariectomized with virgin coconut oil; OVX+T: Ovariectomized with tocotrienol-rich fraction; OVX+VT: Ovariectomized with virgin coconut oil and tocotrienol-rich fraction combination. (a) Significant difference compared to SHAM group, (b) significant difference compared to OVX group.

The sLS/BS of the SHAM group was significantly higher compared to the OVX groups (p<0.05). The sLS/BS of the OVX+E2 and OVX+VT groups were significantly lower compared to the negative control, OVX group (p<0.05) (Figure-5).

### dLS/BS

**Figure-6:**
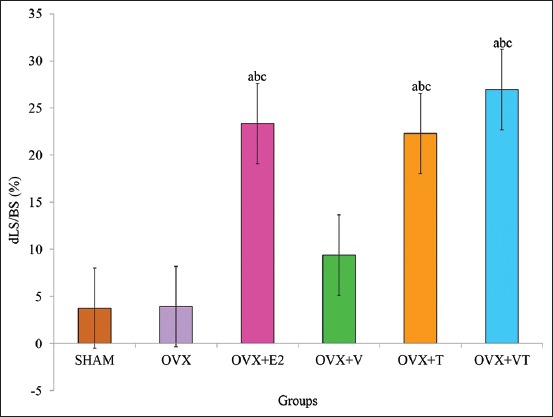
Double-labeled surface/bone surface. Data are expressed as mean±standard error of the mean. SHAM: Sham-operated; OVX: Ovariectomized; OVX+E2: Ovariectomized with Premarin; OVX+V: Ovariectomized with virgin coconut oil; OVX+T: Ovariectomized with tocotrienol-rich fraction; OVX+VT: Ovariectomized with virgin coconut oil and tocotrienol-rich fraction combination. (a) Significant difference compared to SHAM group, (b) significant difference compared to OVX group, (c) significant difference compared to OVX+V group.

The dLS/BS of the OVX+E2, OVX+T and OVX+VT groups was significantly higher compared to the control, SHAM, and negative control, OVX groups (p<0.05) (Figure-6).

### MS/BS

**Figure-7:**
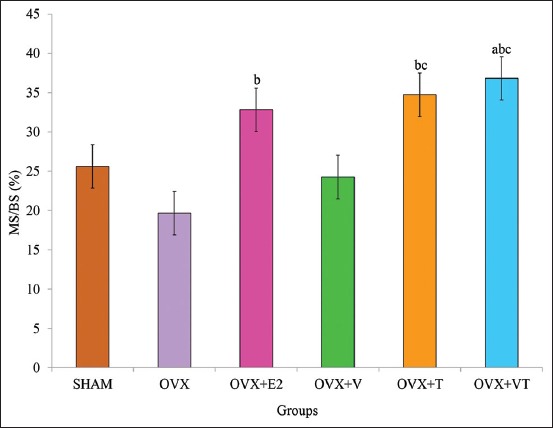
Mineralized surface/bone surface. Data are expressed as mean±standard error of the mean. SHAM: Sham-operated; OVX: Ovariectomized; OVX+E2: Ovariectomized with Premarin; OVX+V: Ovariectomized with virgin coconut oil; OVX+T: Ovariectomized with tocotrienol-rich fraction; OVX+VT: Ovariectomized with virgin coconut oil and tocotrienol-rich fraction combination. (a) Significant difference compared to SHAM group, (b) significant difference compared to OVX group, (c) significant difference compared to OVX+V group.

The MS/BS of the OVX+VT group was significantly higher compared to the control, SHAM group (p<0.05). The MS/BS of the OVX+E2, OVX+T, and OVX+VT groups was significantly higher compared to the negative control, OVX group (p<0.05) (Figure-7).

### MAR

**Figure-8:**
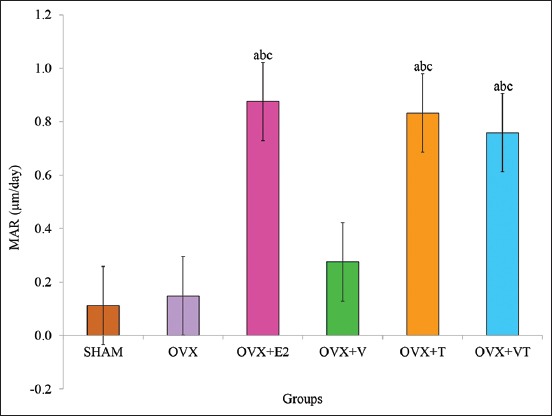
Mineral apposition rate. Data are expressed as mean±standard error of the mean. SHAM: Sham-operated; OVX: Ovariectomized; OVX+E2: Ovariectomized with Premarin; OVX+V: Ovariectomized with virgin coconut oil; OVX+T: Ovariectomized with tocotrienol-rich fraction; OVX+VT: Ovariectomized with virgin coconut oil and tocotrienol-rich fraction combination. (a) Significant difference compared to SHAM group, (b) significant difference compared to OVX group, (c) significant difference compared to OVX+V group.

The MAR of the OVX+E2, OVX+T, and OVX+VT groups was significantly higher compared to the control, SHAM, and negative control, OVX groups (p<0.05) (Figure-8).

### BFR/BS

**Figure-9:**
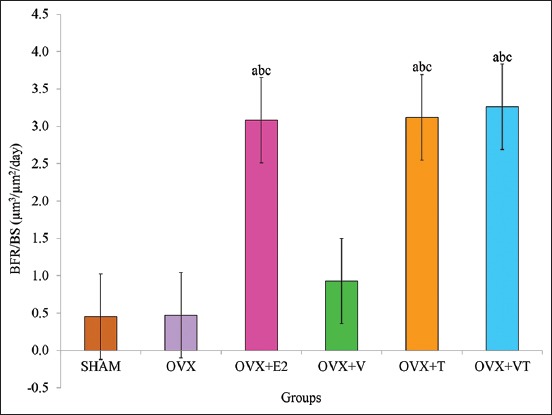
Bone formation rate/bone surface. Data are expressed as mean±standard error of the mean. SHAM: Sham-operated; OVX: Ovariectomized; OVX+E2: Ovariectomized with Premarin; OVX+V: Ovariectomized with virgin coconut oil; OVX+T: Ovariectomized with tocotrienol-rich fraction; OVX+VT: Ovariectomized with virgin coconut oil and tocotrienol-rich fraction combination. (a) Significant difference compared to SHAM group, (b) significant difference compared to OVX group, (c) significant difference compared to OVX+V group.

The OVX+E2, OVX+T, and OVX+VT groups had significant higher BFR/BS compared to the control, SHAM, and negative control, OVX groups (p<0.05) (Figure-9).

### Bone histology of dynamic parameter

**Figure-10:**
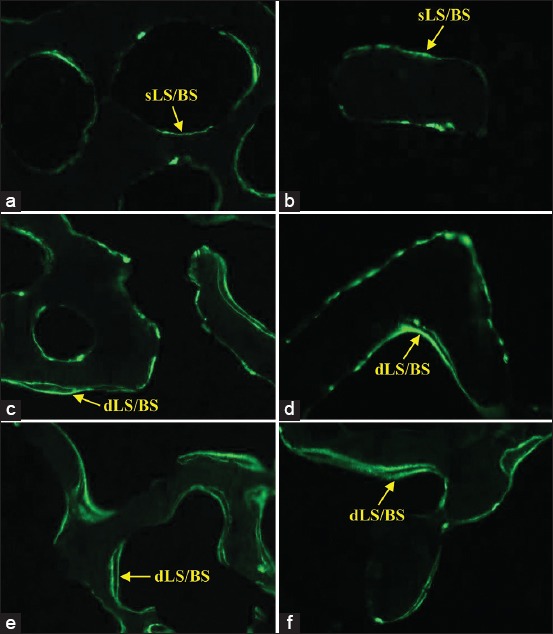
Photomicrographs of the trabecular bone of the distal part of femora labeled with calcein for bone histology of dynamic parameter using fluorescence microscope. Arrows indicated the single-labeled surface/bone surface and double-labeled surface/bone surface 20×. (a) Sham-operated (SHAM), (b) ovariectomized-control (OVX), (c) ovariectomized treated with Premarin (OVX+E2), (d) ovariectomized treated with virgin coconut oil (OVX+V), (e) ovariectomized treated with tocotrienol-rich fraction (OVX+T), (f) ovariectomized treated with virgin coconut oil and tocotrienol-rich fraction combination (OVX+VT).

The photomicrographs of the trabecular bone of the distal part of femora were analyzed using fluorescence microscope. Both the control, SHAM and negative control, OVX groups showed an increase in sLS/BS than dLS/BS while the OVX+E2, OVX+V, OVX+T, and OVX+VT groups had an increase in dLS/BS than sLS/BS (Figure-10).

## Discussion

High cholesterol diet mixed with repeatedly heated palm oil was provided to the OVX rat model to mimic the unhealthy practice of postmenopausal women consuming diets high in cholesterol and containing repeatedly heated palm oil. These conditions would lead to oxidative stress, which may require extra antioxidant supplementations to help the body against free radical attacks [26]. In this study, two forms of well-known antioxidants, VCO and TRF, were used to protect the bone against free-radical induced damage.

Estrogen regulates food intake through the action of leptin, a protein that controls food intake [27]. Leptin level was decreased by ovariectomy [28], resulting in increased food consumption. Estrogen was directly involved in regulation of body weight by binding to estrogen receptors in subcutaneous fat tissue [29]. The result showed that the OVX group had significantly higher mean daily food intake and body weight gain compared to the rest of groups. This was in agreement with the previous reports that OVX rats have higher body weight gain due to fat deposition caused by deficiency of estrogen [30,31]. However, combined or individual supplementations of VCO and TRF to OVX rats were able to reduce the food intake pattern, until they were almost similar to the Premarin group (OVX+E2) and significantly lower than the OVX group. TRF given alone was able to control body weight gain which was comparable to Premarin. The findings on the effects of TRF in reducing body weight gain were similar to previous studies on OVX rats given estrogen [32] and calcium, estrogen, and TRF [33].

OC, a bone formation marker, and CTX, a bone resorption marker, are bone biochemical markers that could detect osteoblast and osteoclast activities, respectively [34,35]. In estrogen deficiency state, the increase in serum CTX levels was associated with the increase in bone turnover rate, leading to bone loss [36]. After 24 weeks of treatment, serum OC levels did not show any significant difference between the groups. This finding was in line with the previous study, which did not find any significant difference in the serum OC levels of groups supplemented with VCO or TRF after an 8-week period of treatment [37].

Hypercholesterolemia was positively correlated with reduction in bone formation and bone density, while bone resorption was increased [38]. The bone mineral density of OVX rats fed high cholesterol diet was significantly decreased after 7 months of treatment [39]. In this study, addition of cholesterol and repeatedly heated palm oil in the diet seemed to promote bone resorption. The result showed that the serum CTX levels were significantly lower in the OVX+E2, OVX+V, OVX+T, and OVX+VT groups compared to the SHAM group. This was in agreement with the previous study, which reported that treatment with anti-osteoporotic agents had decreased the serum CTX level [40]. The post-treatment CTX level in the OVX+VT group was also significantly lower compared to the OVX group. This indicated that VCO-TRF combination was able to reduce the raised CTX level by decreasing bone resorption and bone formation activities. The correlation between serum biomarkers with bone microarchitecture was demonstrated in laboratory animals and postmenopausal women studies [41,42].

Recent clinical diagnostic techniques for osteoporosis were mainly based on using of either X-rays or ultrasound. Both dual X-ray absorptiometry (DXA) and micro-computed tomography have become standard tools to evaluate bone mineral density and bone architecture, respectively. Among the most commonly used techniques, DXA was considered the current gold standard for osteoporosis diagnosis and fracture risk prognosis. However, as a research method, bone histomorphometry supported the interpretation of bone biology [43], developed the potential mechanism of actions of several effective therapies [44] and has been essential in identifying the adverse effects of drugs [45].

Thus, the dynamic bone histomorphometry parameter was used in this study and has been considered the ultimate histomorphometry assessment as it provides a quantitative assessment of the extent of bone formation over a specific period of time. The lower sLS/BS and the higher dLS/BS in both OVX+E2 and OVX+VT groups verified that the addition of Premarin and VCO-TRF supplementation had the potential to overcome the improper bone growth by stimulating bone formation as also seen in the MS/BS, MAR, and BFR/BS parameters. This indicated that VCO-TRF supplementation was as effective as Premarin in producing more newly mineralized bone. In addition, daily VCO-TRF supplementation had also increased the osteoblastic bone formation and decreased osteoclastic bone resorption in the OVX rats. This was indicated in the OVX+VT group by the higher dLS/BS, MS/BS, MAR, and BFR/BS values, and lower sLS/BS values compared to the OVX and OVX+V groups. Previous studies showed Vitamin E supplementation provided a positive effect on bone strength and bone mineral density in animal model studies [46-48].

Therefore, this study discovered that VCO-TRF supplementation on the OVX rats may have additive bone protective effects compared to single supplementation with VCO or TRF that can be beneficial in treating the postmenopausal osteoporosis. This study will help the researchers to uncover the critical areas of antioxidants combination that many researchers were not able to explore. Thus, a new theory on antioxidant therapy may be arrived at combined therapy of VCO and TRF.

## Conclusion

The more superior osteoprotective effects of VCO-TRF supplementation indicated their worthiness as an alternative therapy for the prevention of postmenopausal osteoporosis. Further studies are required to explore their potential as an anti-osteoporotic agent for postmenopausal osteoporosis.

## Authors’ Contributions

FO conceived and designed the study. QMS provided research materials. FH provided logistic support. MMAM conducted research, collected, organized data, wrote the initial and final draft of the article. ANS analyzed, interpreted data, and checked the final draft of the article. All authors read and approved the final manuscript.
